# Supplementary figures and images for: The interplay between vaccination and social distancing strategies affects COVID19 population-level outcomes
Source: PLoS Comput Biol. 2021 Aug 20;17(8):e1009319. doi: 10.1371/journal.pcbi.1009319 (PMC8409608; doi:10.1371/journal.pcbi.1009319)

Proportion of the population

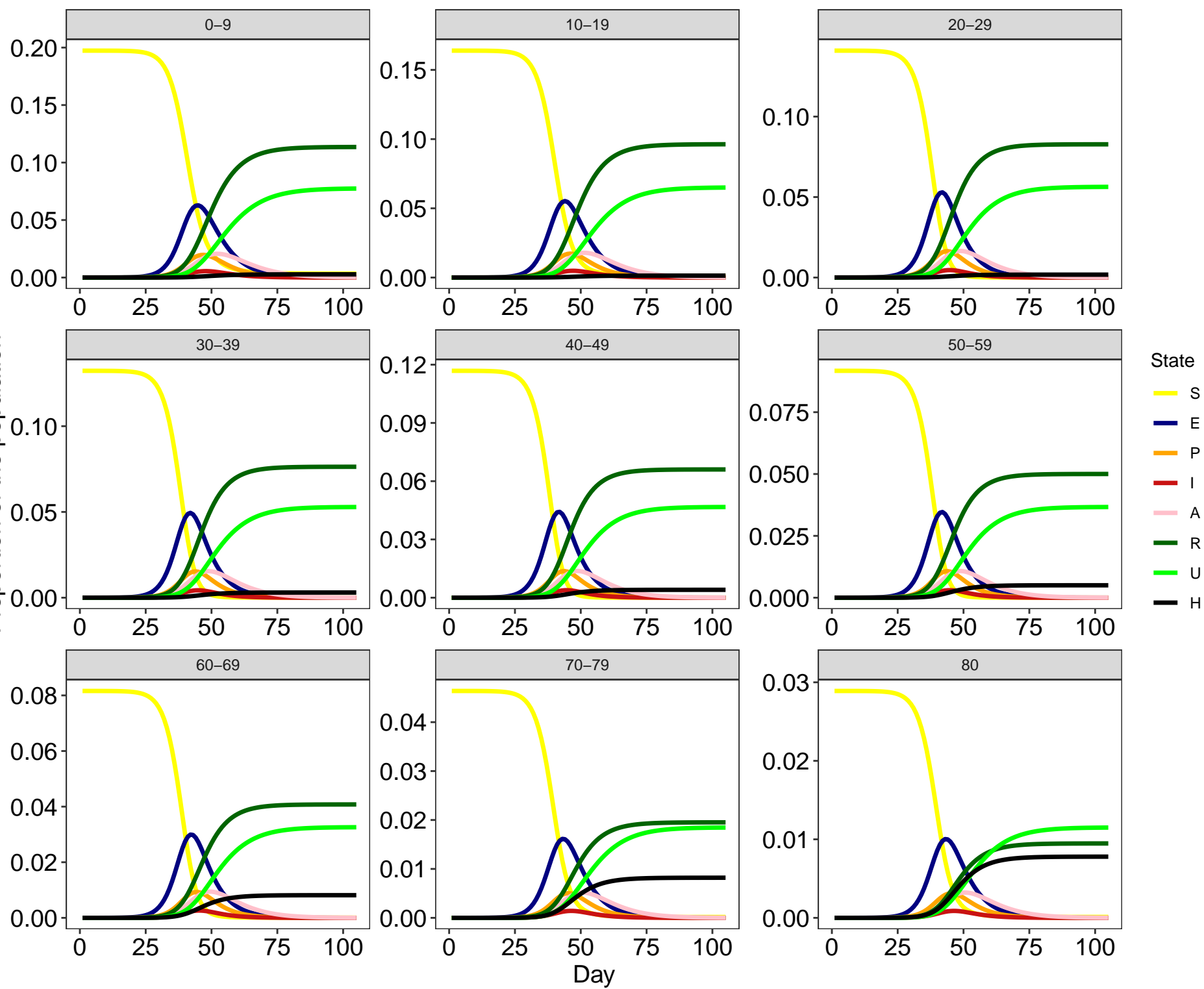

Supplement: S1 Fig — Epidemiological curves for the baseline model, with no vaccination or social distancing. The model follows a population divided to nine age groups as depicted in the figure. S: susceptible; E: exposed; P: presymptomatic; I: infectious and symptomatic; A: infectious and asymptomatic; R: removed to quarantine and then recovered naturally, and immune; U: recovered naturally, and immune; H: hospitalized. (PDF) [file pcbi.1009319.s001.pdf]

Proportion of the population

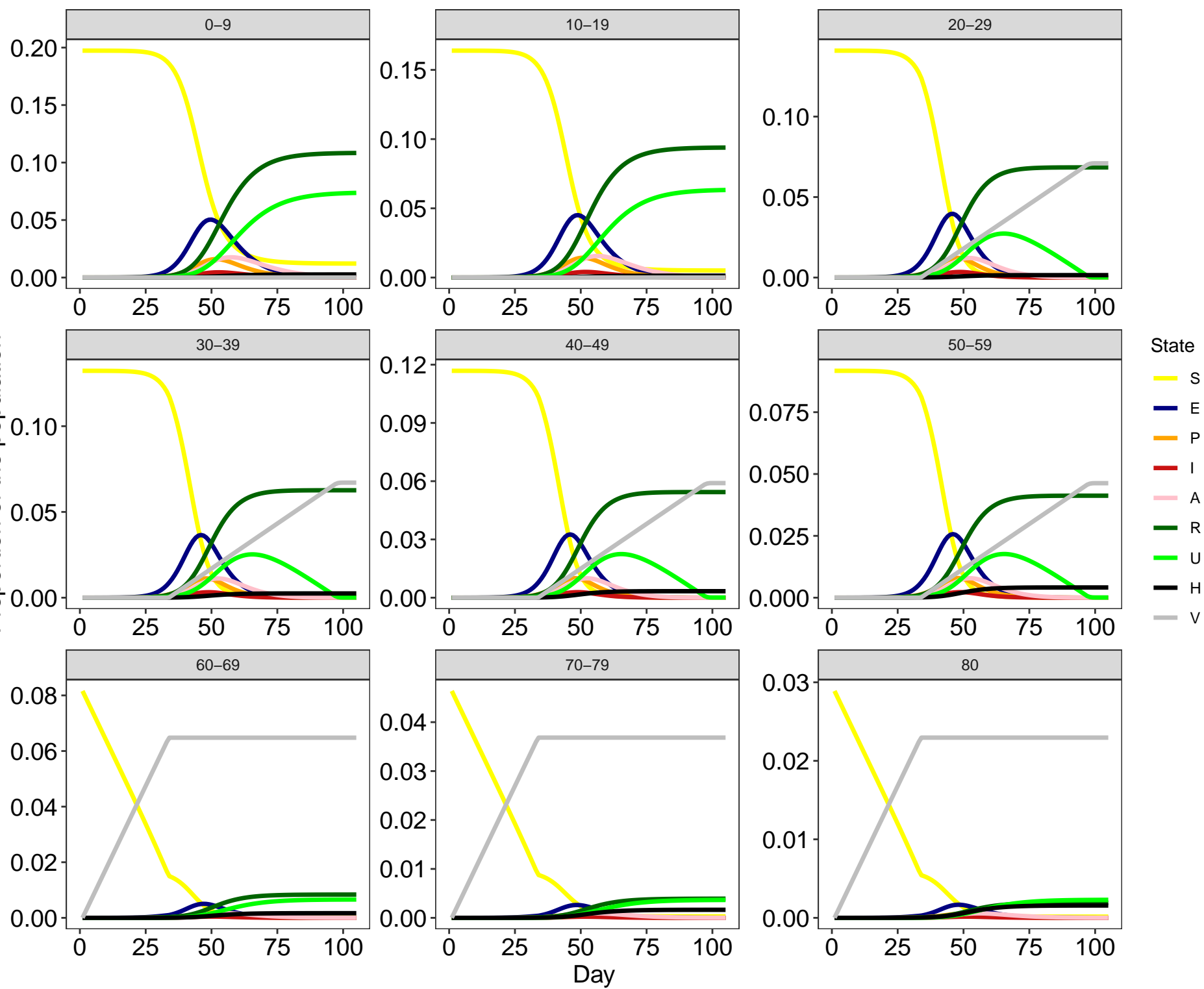

Supplement: S2 Fig — Epidemiological curves with vaccination (κ = 0.2) and no social distancing. The model follows a population divided to nine age groups as depicted in the figure. S: susceptible; E: exposed; P: presymptomatic; I: infectious and symptomatic; A: infectious and asymptomatic; R: removed to quarantine and then recovered naturally, and immune; U: recovered naturally, and immune; H: hospitalized; V: vaccinated. (PDF) [file pcbi.1009319.s002.pdf]

Proportion of the population

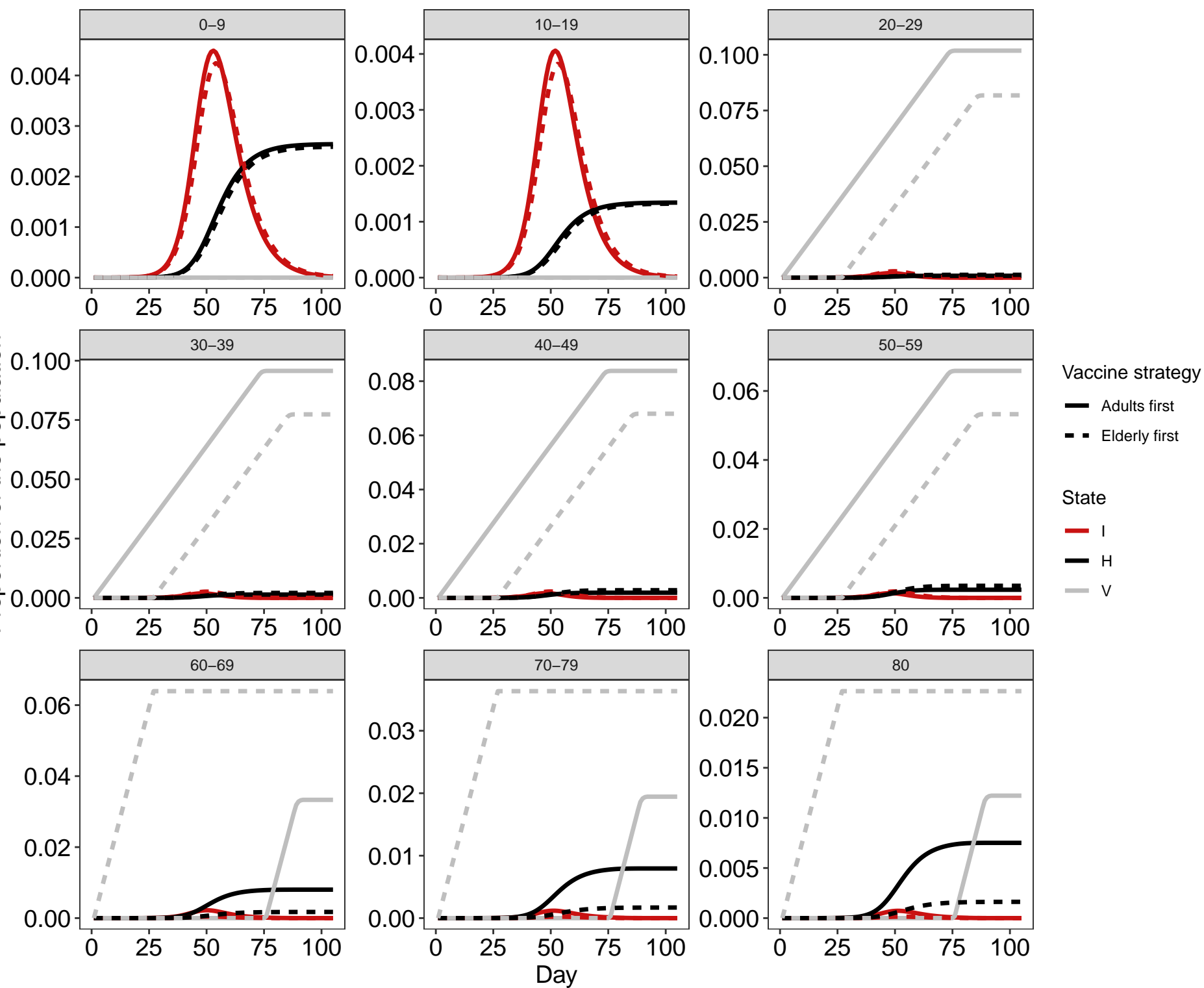

Supplement: S3 Fig — Dashed and solid lines depict two strategies, respectively: (i) vaccinating all elderly (60+) and then all adults (20-59) and (ii) vaccinating all adults and then all elderly. This example is for κ = 0.5 (0.5% of the population is vaccinated per day), with no social distancing. In the first strategy, after about 35 days there are no more elderly to vaccinate and the model shifts to vaccinating adults (gray dashed line). In the second strategy, the switch to vaccinating elderly occurs after about 75 days because the number of adults in the population is larger than that of elderly. Most of the reduction in hospitalizations is obtained when the elderly are prioritized (compare black lines). I: infectious and symptomatic (red); H: hospitalized (black); V: vaccinated (gray). (PDF) [file pcbi.1009319.s003.pdf]

Proportion of the population

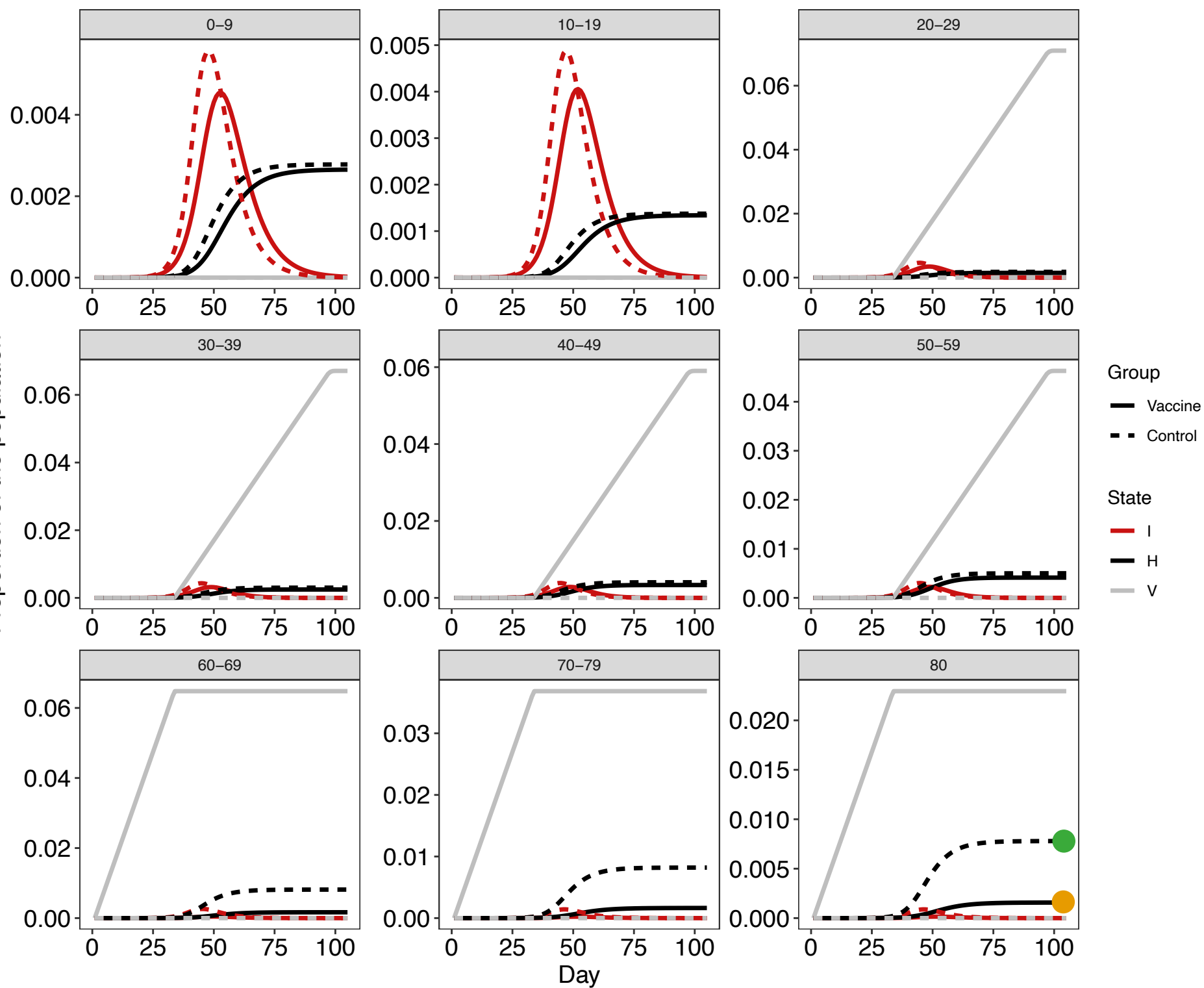

Supplement: S4 Fig — Dashed lines depict control (no vaccine). Solid lines depict a strategy in which we first vaccinate elderly (60+) and then adults (20-59). In this example we use κ = 0.4 (0.4% of the population is vaccinated per day) and there is no social distancing. After about 25 days there are no more elderly to vaccinate and the model shifts to vaccinating adults (gray solid line). It is clear that most of the reduction in hospitalizations is obtained for the elderly groups (compare dashed to solid black lines). The orange and green dots mark the Hj(κ=0.4) and Hj0 values (for j = 80+) used in Eq 1 in the main text, respectively. Model was run for 30 weeks but 12 weeks are plotted here for clarity. I: infectious and symptomatic (red); H: hospitalized (black); V: vaccinated (gray). (PDF) [file pcbi.1009319.s004.pdf]

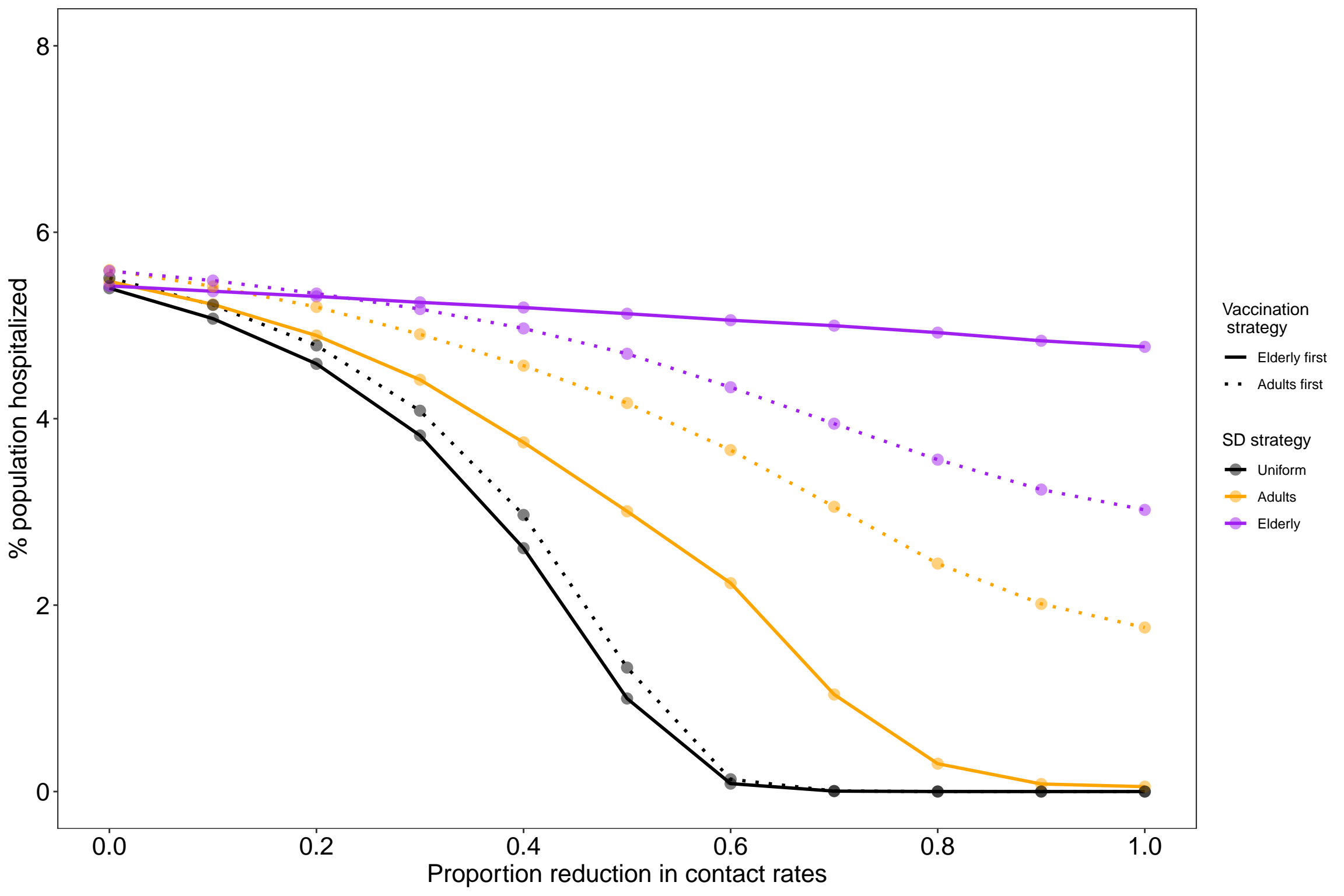

Supplement: S5 Fig — The plot depicts PH (y-axis) as a function of strength of social distancing (x-axis), vaccination rates (κ; line colors), vaccination strategies (columns) and social distancing strategies (rows). (PDF) [file pcbi.1009319.s005.pdf]

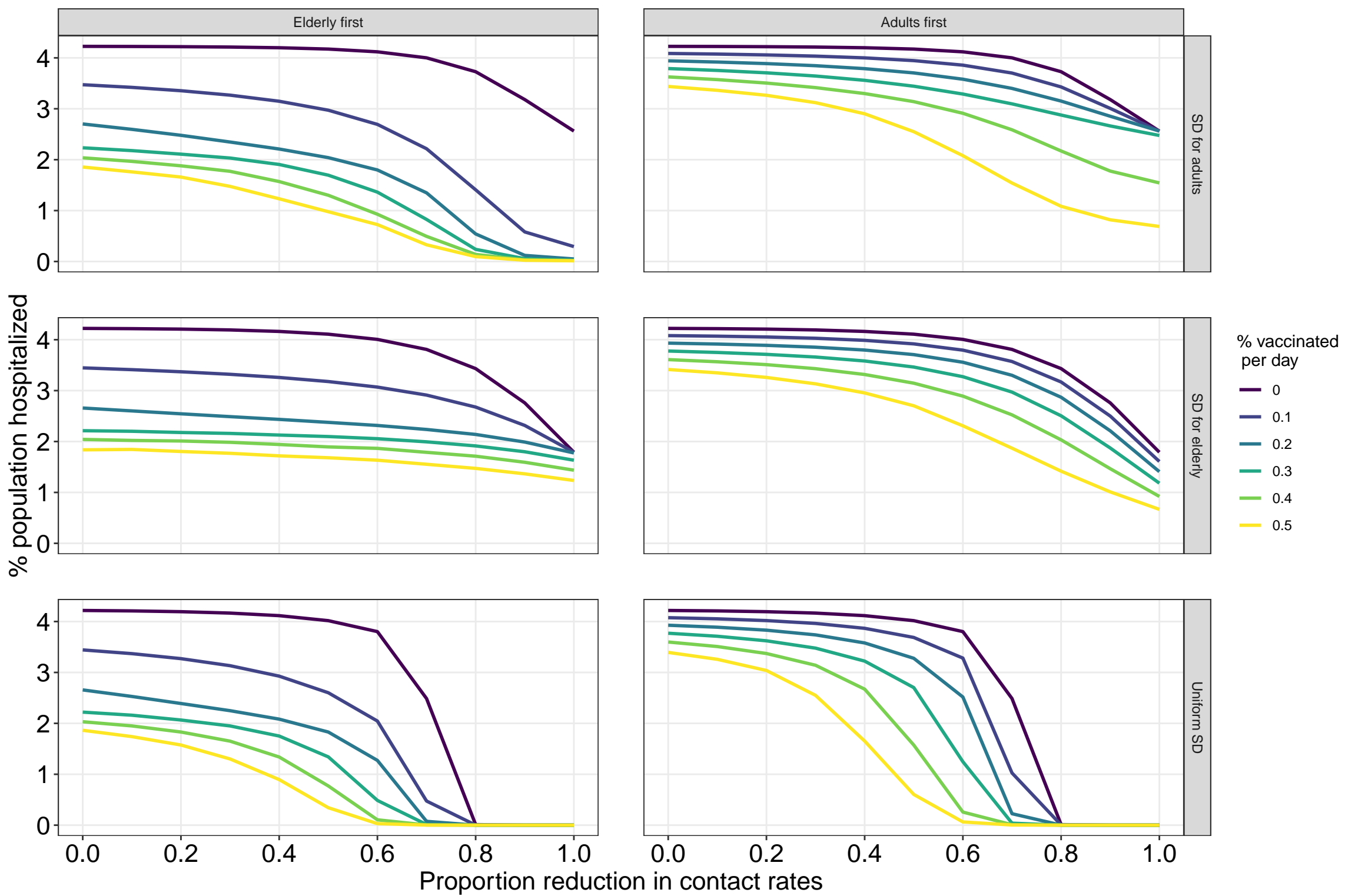

Supplement: S6 Fig — Each data point represents a combination of vaccination strategy (solid vs. dotted lines), social distancing strategy (colors) at particular social distancing strength (x-axis). Simulations were run for daily deployment of κ = 0.5% of the population. The probability of hospitalization was equal to the mean probability across ages (i.e., mean of hj). (PDF) [file pcbi.1009319.s006.pdf]

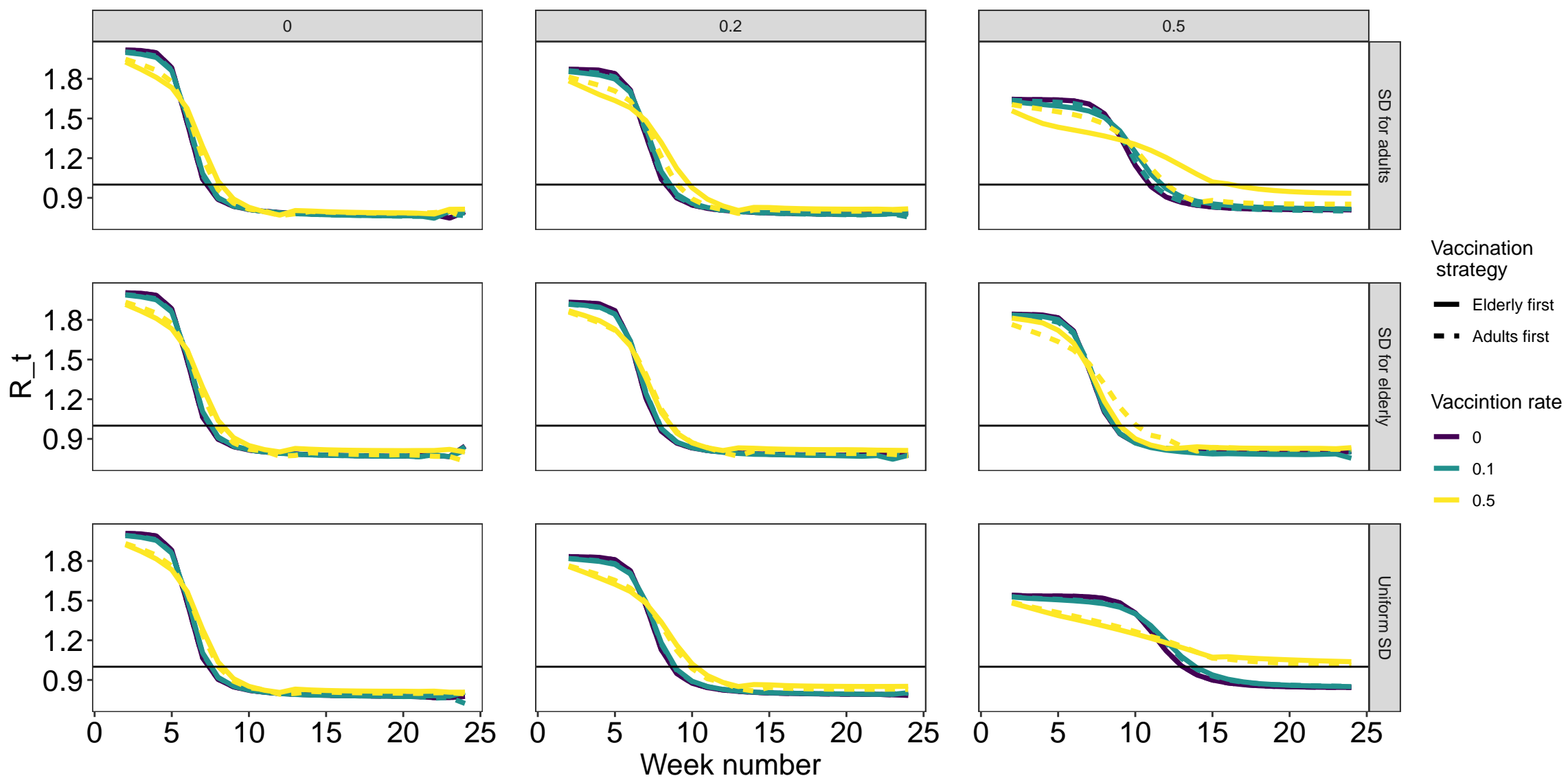

Supplement: S8 Fig — Columns represent three levels of proportion of reduction in contact rates: 0 (no reduction), 0.2, and 0.5. Each row is a social distancing strategy. Rt was calculated for 7-day periods as descried in the main text. (PDF) [file pcbi.1009319.s008.pdf]

**A**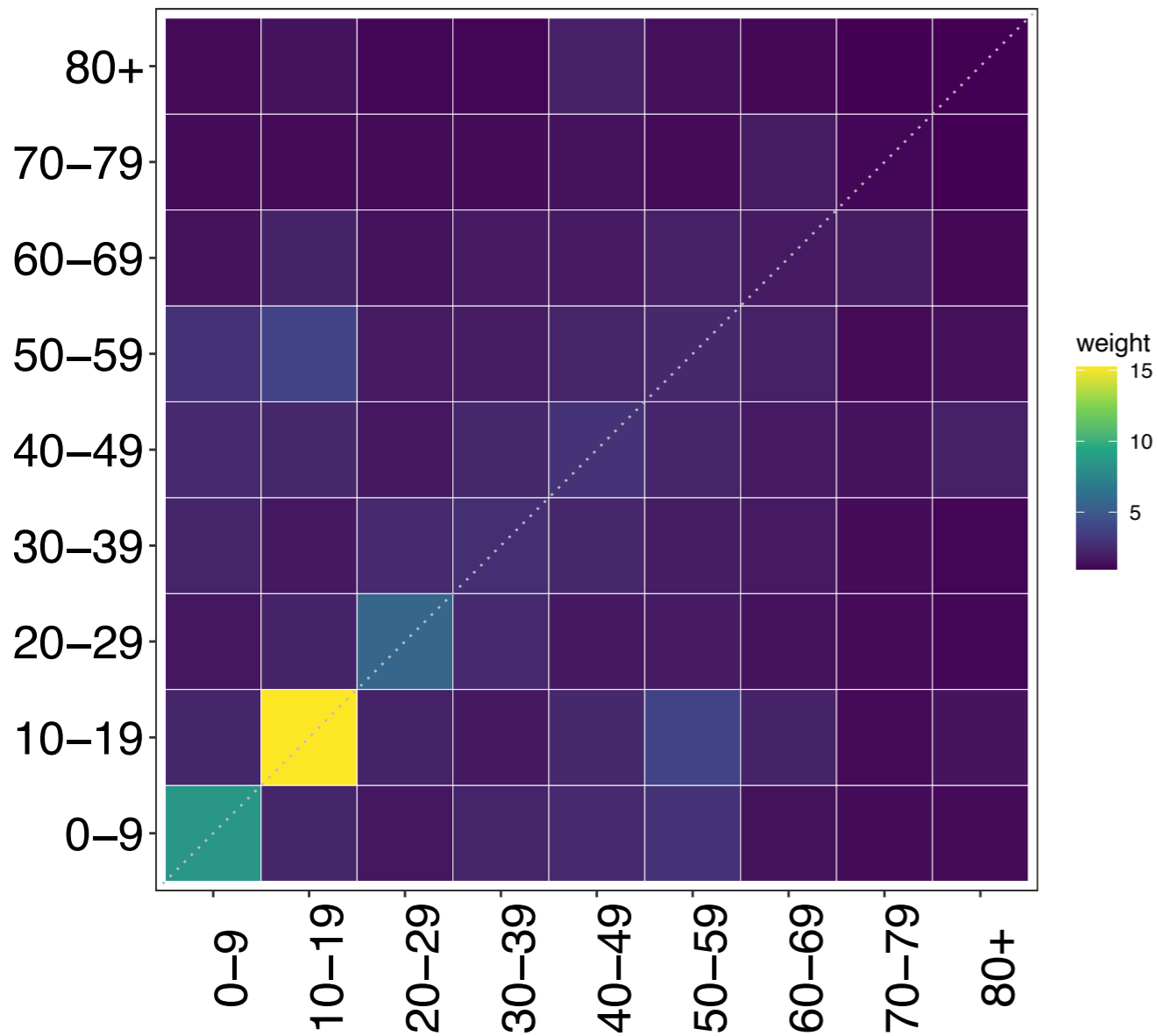**B**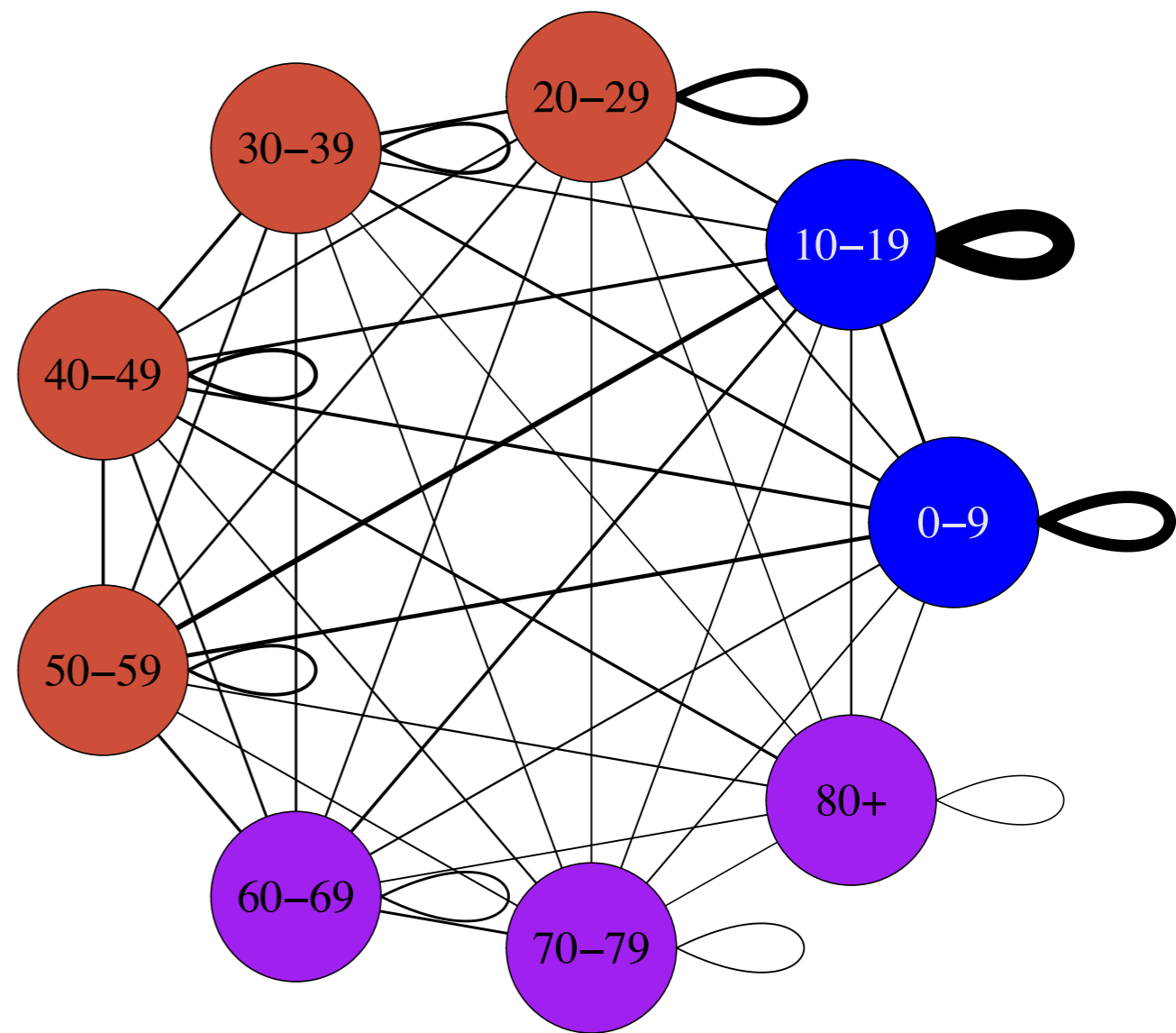

Supplement: S9 Fig — (A) Contact matrix for Italy calculated from data collected by Mossong et al. (2008). Matrix cells depict the mean number of daily contacts between people in different age groups. Diagonal cells depict contacts between individuals from the same age group. (B) The same data as in (A), represented as a network of contacts. Edge widths depict contact rates. (PDF) [file pcbi.1009319.s009.pdf]
