# Supplementary material for: The interplay between vaccination and social distancing strategies affects COVID19 population-level outcomes
Source: PLoS Comput Biol. 2021 Aug 20;17(8):e1009319. doi: 10.1371/journal.pcbi.1009319 (PMC8409608; doi:10.1371/journal.pcbi.1009319)
Supplement: S7 Fig — Columns and rows represent social distancing and vaccination strategies, respectively. This example is for a 50% reduction in contacts. The dashed lines mark the total number of hospitalizations for a κ = 0.5 vaccination rate. For example, under the adult social distancing scenario, Ha = 219, 882 and He = 84, 321. (PDF) [file pcbi.1009319.s007.pdf]

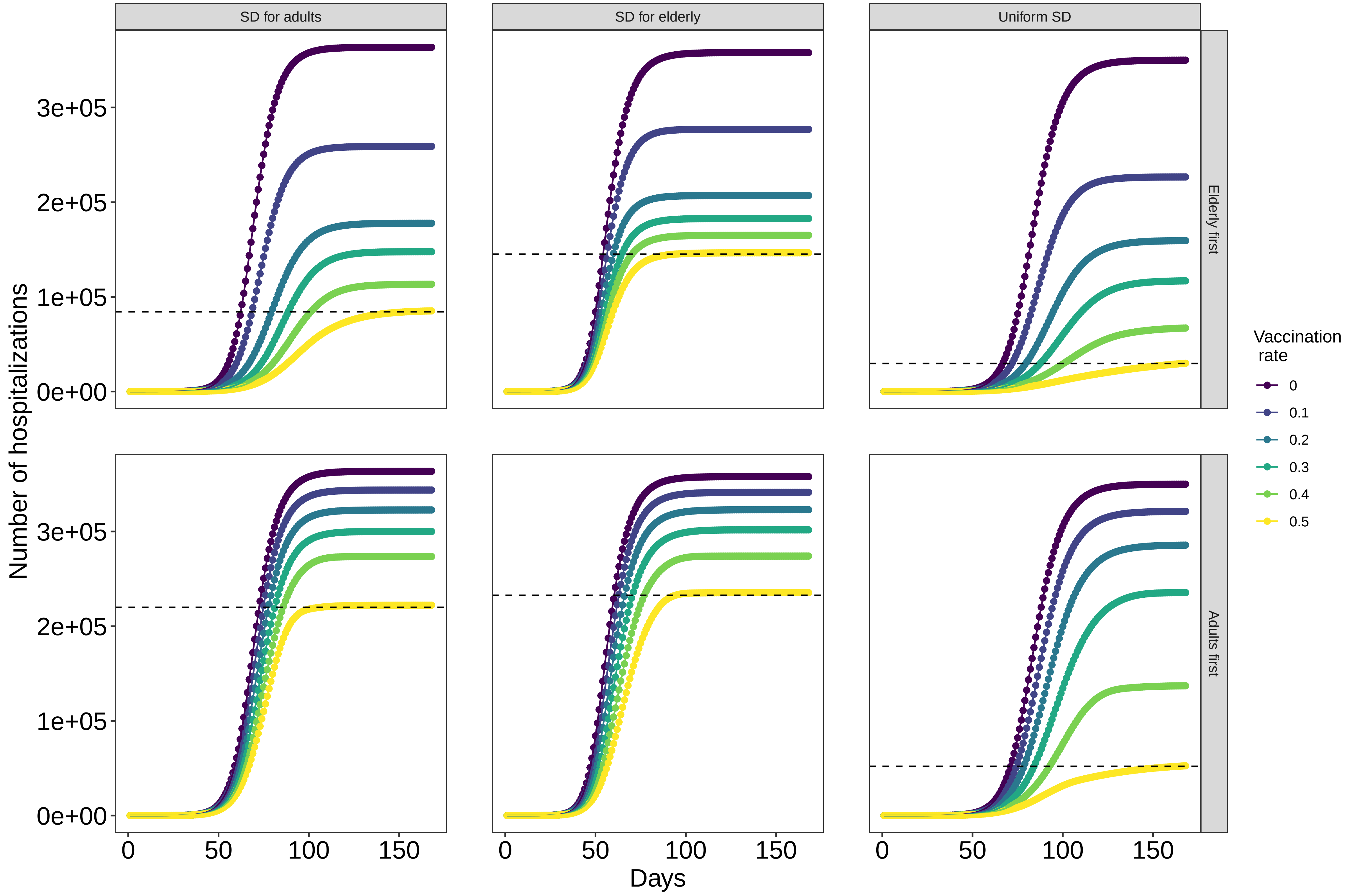
SD for adults
SD for elderly
Uniform SD
Elderly first
Adults first
Number of hospitalizations
Days
0e+00
1e+05
2e+05
3e+05
0
50
100
150
Vaccination rate
0
0.1
0.2
0.3
0.4
0.5
