# Supplementary material for: The interplay between vaccination and social distancing strategies affects COVID19 population-level outcomes
Source: PLoS Comput Biol. 2021 Aug 20;17(8):e1009319. doi: 10.1371/journal.pcbi.1009319 (PMC8409608; doi:10.1371/journal.pcbi.1009319)
Supplement: S1 Table — (PDF) [file pcbi.1009319.s010.pdf]

Table S1. Population structure of Israel and relevant parameters. j is the ID of the age group; Proportion is the proportion of the age group in the Israeli population; h_j is the probability of hospitalization taken from [1]. We preferred this data source because of its high resolution and high number of cases that provides better accuracy for calculating proportions. This probability is not expected vary greatly between countries as age-dependent hospitalization has a strong biological rather than social component in countries which are culturally similar. Indeed, we contrasted it with Israeli data from [2] and proportions were highly similar.

j	Age	Proportion	h_j
1	0-9	0.20	0.024
2	10-19	0.16	0.014
3	20-29	0.14	0.021
4	30-39	0.13	0.038
5	40-49	0.12	0.058
6	50-59	0.09	0.091
7	60-69	0.08	0.166
8	70-79	0.05	0.296
9	80+	0.03	0.451

References

1. Florida Department of Health. Florida Department of Health Open Data;. `https://open-fdoh.hub.arcgis.com/datasets/florida-covid19-case-line-data-3/data`.

2. Israel Ministry of Health. Israel Corona Dashboard;. `https://datadashboard.health.gov.il/COVID-19/general`.
