# Supplementary material for: The interplay between vaccination and social distancing strategies affects COVID19 population-level outcomes
Source: PLoS Comput Biol. 2021 Aug 20;17(8):e1009319. doi: 10.1371/journal.pcbi.1009319 (PMC8409608; doi:10.1371/journal.pcbi.1009319)
Supplement: S2 Table — (PDF) [file pcbi.1009319.s011.pdf]

Table S1. Study parameters. The proportion of the target population accepting vaccination was applied computationally and does not appear in the model equations. The values noted are those used in the main simulation (experiment 1). We also conducted sensitivity analyses. The complete set of experiments that includes all values for all parameters can be found in the GitHub repository (file `experiments.csv`).

Parameter	Type	Value	Description	Reference
β	rate	0.31	infections per person per day	
α	rate	1/6.4 days	incubation time	[1]
γ	rate	1/7 days	recovery rate	[2]
ϕ	rate	1/2.1 days	time in presymptomatic stage	[3]
η	rate	1 day	time to quarantine upon showing symptoms	
e	proportion	0.95	vaccine efficiency	[4]
b	proportion	0.5	relative infectiousness of presymptomatic and asymptomatic relative to symptomatic	[5]
h	proportion	Table S1	Proportion of cases hospitalized	[6, 7]
m	proportion	0.4	proportion of asymptomatic infections	[8]
p	proportion	0.8	proportion of target population accepting vaccines	[9]

References

1. Backer JA, Klinkenberg D, Wallinga J. Incubation period of 2019 novel coronavirus (2019-nCoV) infections among travellers from Wuhan, China, 20-28 January 2020. Euro Surveill. 2020;25(5). doi:10.2807/1560-7917.ES.2020.25.5.2000062.

2. Cheng HY, Jian SW, Liu DP, Ng TC, Huang WT, Lin HH, et al. Contact Tracing Assessment of COVID-19 Transmission Dynamics in Taiwan and Risk at Different Exposure Periods Before and After Symptom Onset. JAMA Intern Med. 2020;180(9):1156–1163. doi:10.1001/jamainternmed.2020.2020.

3. Davies NG, Klepac P, Liu Y, Prem K, Jit M, CMMID COVID-19 working group, et al. Age-dependent effects in the transmission and control of COVID-19 epidemics. Nat Med. 2020;26(8):1205–1211. doi:10.1038/s41591-020-0962-9.

4. Polack FP, Thomas SJ, Kitchin N, Absalon J, Gurtman A, Lockhart S, et al. Safety and Efficacy of the BNT162b2 mRNA Covid-19 Vaccine. N Engl J Med. 2020;383(27):2603–2615. doi:10.1056/NEJMoa2034577.

5. Subramanian R, He Q, Pascual M. Quantifying asymptomatic infection and transmission of COVID-19 in New York City using observed cases, serology, and testing capacity. Proc Natl Acad Sci U S A. 2021;118(9). doi:10.1073/pnas.2019716118.

6. Florida Department of Health. Florida Department of Health Open Data;. `https://open-fdoh.hub.arcgis.com/datasets/florida-covid19-case-line-data-3/data`.

7. Israel Ministry of Health. Israel Corona Dashboard;. https://datadashboard.health.gov.il/COVID-19/general.

8. Lavezzo E, Franchin E, Ciavarella C, Cuomo-Dannenburg G, Barzon L, Del Vecchio C, et al. Suppression of a SARS-CoV-2 outbreak in the Italian municipality of Vo'. Nature. 2020;584(7821):425–429. doi:10.1038/s41586-020-2488-1.

9. Dror AA, Eisenbach N, Taiber S, Morozov NG, Mizrachi M, Zigron A, et al. Vaccine hesitancy: the next challenge in the fight against COVID-19. Eur J Epidemiol. 2020;35(8):775–779. doi:10.1007/s10654-020-00671-y.
